# CYP1A1 Relieves Lipopolysaccharide-Induced Inflammatory Responses in Bovine Mammary Epithelial Cells

**DOI:** 10.1155/2018/4093285

**Published:** 2018-02-28

**Authors:** Wen-Yao Zhang, Hao Wang, Shaopei Qi, Xixi Wang, Xueru Li, Kun Zhou, Yong Zhang, Ming-Qing Gao

**Affiliations:** ^1^College of Veterinary Medicine, Northwest A&F University, Yangling, Shaanxi 712100, China; ^2^Innovation Experimental College, Northwest A&F University, Yangling, Shaanxi 712100, China; ^3^Key Laboratory of Animal Biotechnology, Ministry of Agriculture, Northwest A&F University, Yangling, Shaanxi 712100, China

## Abstract

The expression of cytochrome P4501A1 (CYP1A1) enzyme is changed in various organs during the host response to inflammation or infection, leading to alterations in the metabolism of endogenous and exogenous compounds. Results of this study showed that CYP1A1 expression was significantly downregulated in the mammary tissue of bovine with mastitis, in inflammatory epithelial cells (INEs) extracted from the tissue, and in lipopolysaccharide- (LPS-) induced INEs compared with their corresponding counterparts. Overexpression of CYP1A1 in bovine mammary epithelial cells alleviated the LPS-induced inhibition of epithelial proliferation, abated the LPS-induced increase of gene expression and protein secretion of inflammatory cytokine tumor necrosis factor-*α* and interleukin-6, and attenuated the LPS-induced activation of NF-*κ*B signaling. These findings suggest that CYP1A1 has immense potential in the regulation of inflammatory responses in bovine mammary epithelial cells during mastitis and may serve as a useful therapeutic target in mitigating injuries caused by inflammatory overreaction.

## 1. Introduction

The mammary gland epithelium functions as a milk-secreting organ and as a primary and selective barrier against pathogens [1]. As a part of the immune system, it maintains a predominant role in pathogen-induced bovine mastitis and mediates the innate and adaptive immune responses of the mammary gland by secreting proinflammatory cytokines and chemokines, including tumor necrosis factor- (TNF-) *α*, interleukin- (IL-) 1*β*, and IL-6, or promoting T and B lymphocyte responses [2–4]. *Escherichia coli* is an important pathogen causing bovine mastitis. Lipopolysaccharide (LPS), the major cell membrane component of *E. coli*, can specifically bind toll-like receptors and induce inflammation responses, which are predominantly mediated by the activation of the NF-*κ*B signaling pathway. This cascade of events induces the overproduction of proinflammatory cytokines [3, 5, 6]. Uncontrolled inflammatory responses can be harmful, even fatal, to the host [7]. Therefore, alleviating the production of these proinflammatory mediators is highly important to prevent excessive inflammatory injury to the mammary gland [8].

Cytochrome P4501A1 (CYP1A1) of the CYP family can be detected in many tissues, including breast epithelium [9, 10]. Infections or inflammatory stimuli can alter the expression of CYP1A1 in various organs of humans and animals [11]. CYP1A1 is activated in hepatocytes incubated with serum from rabbits and humans with inflammatory reaction [12]. It also mediates the inflammatory reaction in human bronchial epithelial cells induced by diesel exhaust particles [13]. The differential expression of CYP family members, including CYP1A1, is significant in LPS-induced hepatitis [14]. CYP1A1 is downregulated in astrocytes during brain inflammation [15]. As a target regulated by CYP1A1, PPAR-*γ* inhibits the expression of inflammatory activating factors along with NF-*κ*B [16], and increased CYP1A1 expression can be repressed by inhibition of NF-*κ*B [17]. These studies strongly suggested that understanding the connection between CYP1A1 and the NF-*κ*B signaling pathway is necessary to develop effective strategies for the treatment of inflammation-associated diseases.

Considering the growing body of evidence showing that CYP1A1 is involved in inflammation-related diseases and our preliminary finding from a high-throughput sequencing analysis that CYP1A1 expression is suppressed in LPS-treated epithelial cells, we hypothesized that CYP1A1 also participates in the biological process of bovine mammary epithelial cells during bovine mastitis. To confirm this speculation, CYP1A1 expression was first examined in the mammary tissue of bovine with or without mastitis, extracted epithelial cells from these two types of tissues, and treated mammary epithelial cells with LPS in this study. The functions and potential mechanism of CYP1A1 in proliferation and the inflammatory cytokine secretion in epithelial cells induced by LPS were further clarified using an in vitro epithelial cell model of bovine mastitis.

## 2. Materials and Methods

### 2.1. Cell Culture

Mammary alveolar cell-T (MAC-T), a continuous cell line derived from bovine mammary tissue by stable transfection with the SV40 large T-antigen, was provided by Professor Mark D. Hanigan (Virginia Polytechnic Institute and State University, Blacksburg, VA). Inflammatory epithelial cells (INEs) extracted from bovine mammary glands with clinical mastitis and normal epithelial cells (NEs) from dairy cows slaughtered because of fractured legs during lactation were isolated by our research team in a previous research [18]. The mammary tissues were grossly examined by an experienced anatomical pathologist and subsequently histopathologically examined to distinguish mastitic and normal tissues. Bacterial contamination of all obtained tissue was determined by culturing 10 *μ*L of residual milk from the mammary tissue samples on blood agar plates that were sent to the Microbiology Experimental Center of Yangling Demonstration Zone Hospital (Yangling, Shaanxi, China) by using standard bacteriological techniques [18]. Tissues from dairy cows with mastitis caused by *E. coli* were used in this study.

MAC-T cells, INEs, and NEs were cultured in complete DMEM/F12 medium (Gibco BRL, Burlington, ON) supplemented with 10% fetal bovine serum, 100 IU/mL penicillin, and 100 *μ*g/mL streptomycin (Gibco BRL, Grand island, NY) at 37°C in an incubator with 5% CO_2_. The cells were passaged by digestion with 0.15% trypsin and 0.02% EDTA.

### 2.2. High-Throughput Sequencing Analysis

cDNA libraries were constructed using RNA samples extracted from mammary epithelial cells with or without LPS treatment in accordance with a previously described procedure [18]. The library products were sequenced using Illumina HiSeq™ 2000 (Beijing Genomics Institute, Shenzhen, China) to screen the differential expression genes that may function in bovine mammary epithelial cells during LPS-induced infection.

### 2.3. RNA Extraction and Real-Time Quantitative PCR (RT-qPCR)

Before the isolation of total RNA, the cells were washed twice with PBS. Ice-cold TRIzol solution (TransGene, Shanghai, China) was used to resuspend and lyse the cells. Total mRNA extraction was executed using the RNeasy Kit (TransGene). The concentration of total mRNA was measured from the absorbance at 260/280 nm with a spectrophotometer (NanoDrop Technologies, Wilmington, DE), and total mRNA was used directly for cDNA synthesis with the TransScript II First-Strand cDNA Synthesis SuperMix (TransGene).

The design of all primers was based on sequences from the National Center for Biotechnology Information Database. The specificity of the primers was tested using Primer-BLAST. The primers are listed in Additional File 2. The PCR primers were synthesized from Sangon Biotech (Shanghai, China). Quantitative detection of gene expression was executed with an iQ5 light cycler (Bio-Rad, Hemel Hempstead, UK) by using TransStart Probe qPCR SuperMix (TransGene) in 20 *μ*L reaction systems containing 1 *μ*mol/L forward and reverse primers for each gene (Sangon Biotech). The primer specificities were estimated on the basis of melt curves. Reactions were conducted in triplicate, and the data were expressed as relative gene expression = 2^−ΔΔCt^. The amount of each target gene was normalized to GAPDH mRNA, and the relative quantification algorithm was employed to quantify normalized target gene expression compared with the control condition (untreated cells, normal tissue, or empty vector), which was set to 1.

### 2.4. Molecular Cloning of Full-Length Bovine CYP1A1 and Construction of CYP1A1 Overexpression Plasmids

Primers for the molecular cloning of full-length bovine CYP1A1 gene were designed based on sequences from the National Center for Biotechnology Information Database. The forward primer was 5′-CTAGCTAGCGGATCATGTTTTCTGTGTTTGGAC-3′, and the reverse primer was 5′-GCGGCCGCCTAAGAGCGCATGTGCGCCTGAAAG-3′. The full-length CYP1A1 cDNA was obtained from bovine MAC-T cDNA by performing a two-step PCR as follows: activation at 95°C for 1 min; 32 cycles of denaturation at 95°C for 20 s, annealing at 57°C for 20 s, and extension at 72°C for 1 min; and a final extension at 72°C for 5 min. The PCR products were separated using 1% sepharose gel and collected using a StarPrep Gel Extraction Kit (GenStar BioSolutions, Beijing, China). Then, the collected cDNA was added with polyA at 72°C for 30 min by using a DNA A-Tailing Kit (TakaRa, Dalian, China). The product with added polyA was further collected using a StarPrep Gel Extraction Kit (GenStar BioSolutions) and connected to a pMD19-T vector (TakaRa) by using T4 DNA Ligase (TakaRa) at 4°C for 12 h. Finally, the plasmids from the transformants of Trans5*α* containing the sequence for the molecular cloning of full-length bovine CYP1A1 gene were collected using a TIANprep Rapid Mini Plasmid Kit (TIANGEN, Shanghai, China) and sequenced by BIG Tech (Shenzhen, China) to verify the correct DNA sequence.

The plasmid of the pMD19-T vector connected with the correct sequence was digested by restriction enzymes Nhel and Notl (New England Biolabs, Ipswich, MA). The enzyme-digested products were separated using 1% sepharose gel, and the target band of CYP1A1 was collected using the StarPrep Gel Extraction Kit (GenStar BioSolutions). To efficiently transfect the plasmid into the epithelium, the widely used lentiviral expression vector pCDH-CMV-MCS-EF1-copGFP-T2A-Puro plasmid (SBI plasmid, CD513B-1) was chosen as the basic target plasmid, and the full-length bovine CYP1A1 cDNAs were cloned into this target plasmid. After undergoing transformation, the transformants were cultured in a 100 mg/mL ampicillin LB selection medium. The plasmids were extracted from selected transformants by using a TransGene Plasmid MaxiPrep Kit (TransGene) and then validated with a two-step PCR. The empty basic target plasmid was used as the control. Finally, the target vector was transfected into MAC-T cells through electroporation. The expression of the full-length bovine CYP1A1 gene was confirmed through Western blot and RT-PCR after transfection for 48 h.

### 2.5. Cell Transient Transfection and Treatment with LPS

MAC-T cells, growing exponentially on 6 cm dishes, were digested with 0.15% trypsin and 0.02% EDTA and washed twice with Opti-MEM (Gibco). Then, the MAC-T cells were resuspended in an electroporation buffer and mixed with 10 *μ*g of CYP1A1 overexpression plasmid, blank plasmid, or no plasmid. After incubation for 10 min at 25°C, the MAC-T cells were resuspended again and devolved into BTX electroporation cuvettes (BTX lnc., San Diego, CA). The electroporation cuvettes were electroporated at 510 V with three pulses of 1 ms duration using the BTX electro-cell manipulator ECM2001 (BTX lnc.). The electroporated cells were balanced for 10 min at 4°C and then plated on 6 cm plates at 1 × 10^6^ cells per plate. After incubation for 12 h at 37°C in a humidified 5% CO_2_ incubator, the MAC-T cell medium was replaced with fresh complete medium and then the culture was continued for 12 h. Then, the medium was replaced with a complete medium containing LPS at a final concentration of 10 ng/*μ*L. After stimulation for 3 h with LPS, the MAC-T cell culture supernatants were discarded and replaced with a fresh medium. After another incubation for other 24 h, the MAC-T cells were used in subsequent experiments.

### 2.6. Western Blot

Protein lysates from the MAC-T cells were prepared with ice-cold PRO-PREP Protein Extraction Solution (iNtRON Biotechnology, Inc., Gyeonggi-do, Korea) in accordance with the manufacturer's instructions. Lysates were precipitated at 1200 rpm for 10 min at 4°C. The total protein content was determined using the Bradford Easy Protein Quantitative Kit (TransGene). Equal amounts of protein extracts in lysis buffer were separated using 4%–12% polyacrylamide gels (German, Sigma-Aldrich) and then transferred onto a nitrocellulose membrane. Resolved proteins were blotted onto PVDF transfer membranes (Millipore Co., Bedford, MA) and then blocked with 10% nonfat milk in TBST. The membranes were incubated with anti-CYP1A1 (Sangon Biotech, Shanghai, China), anti-NF-*κ*B (Bioss, Beijing, China), anti-phospho-NF-*κ*B (Santa Cruz Biotechnology lnc., Santa Cruz, CA, USA), and anti-GAPDH (TransGene) antibodies at 4°C overnight. The membranes were washed three times with TBST for 5 min before incubation with HRP-conjugated secondary antibodies. Finally, the immunoreactive proteins were visualized using an enhanced chemiluminescence detection kit (Beyotime).

### 2.7. ELISA

The secretion of TNF-*α* and IL-6 in the medium was measured using ELISA. The MAC-T cells and transfected MAC-T cells by the empty vector or CYP1A1 overexpression vector were cultured in a fresh serum-free medium for another 24 h after being treated with LPS for 3 h. Then, the medium was collected, and cell debris was removed via centrifugation. The levels of TNF-*α* and IL-6 secreted in the medium were measured using corresponding ELISA kits (Huzhen Biological Technology Co. Ltd., Shanghai, China) in accordance with the manufacturer's instructions.

### 2.8. Luciferase Assays

Triplicates of 3 × 10^4^ MAC-T cells/well were seeded in 48-well plates and cultured for 12 h. The medium was replaced with a fresh serum-free medium and incubated for another 6 h. The MAC-T cells were cotransfected with CYP1A1 overexpression plasmids, NF-*κ*B luciferase plasmids, and its control vector of Renilla luciferase vector (pRL-TK). After transfecting for 12 h, the MAC-T cells were treated with 10 ng/*μ*L LPS for 3 h at 37°C in a humidified incubator. The cells were cultured in a serum-free medium after LPS treatment, and then luciferase activity was measured using the Dual-Luciferase Reporter Assay System (TransGene) and the Turner BioSystems 20/20 Luminometer.

### 2.9. Cell Proliferation Assay

Cell proliferation was examined using the Cell Counting Kit-8 (CCK-8, Beyotime) in accordance with the manufacturer's protocol. The CYP1A1-transfected MAC-T cells treated with LPS were routinely harvested and seeded into 96-well plates with 100 *μ*L of complete medium at a density of approximately 2 × 10^3^ cells/well. After incubation for 12 h, the complete medium was replaced with a fresh medium. The MAC-T cells were then cultured in a complete medium for 0, 1, 2, and 3 days. Before the MAC-T cell proliferation assay, the medium was replaced with 100 *μ*L of fresh medium containing 10 *μ*L of CCK-8 solution. The cells were then incubated for another 3 h at 37°C in a humidified incubator. At a designated time point, the absorbance of the samples in triplicate wells was measured at 450 nm wavelength with a microplate reader (Bio-Rad, Hercules, CA), and the cell numbers were calculated in reference to a standard curve obtained under the same experimental conditions. The MAC-T cells transfected with the empty vector were used as a control.

### 2.10. Statistical Analysis

Results were reported as mean ± SD. All data were obtained from at least three independent experiments. All statistical analyses were performed using ANOVA (SPSS 11.5, IBM Corporation, Armonk, NY). Statistically significant difference was considered at *p* < 0.05.

## 3. Results

### 3.1. Expression of CYP1A1 Was Downregulated in Bovine Mammary Tissue and Epithelial Cells under Inflammatory Conditions

**Figure 1:**
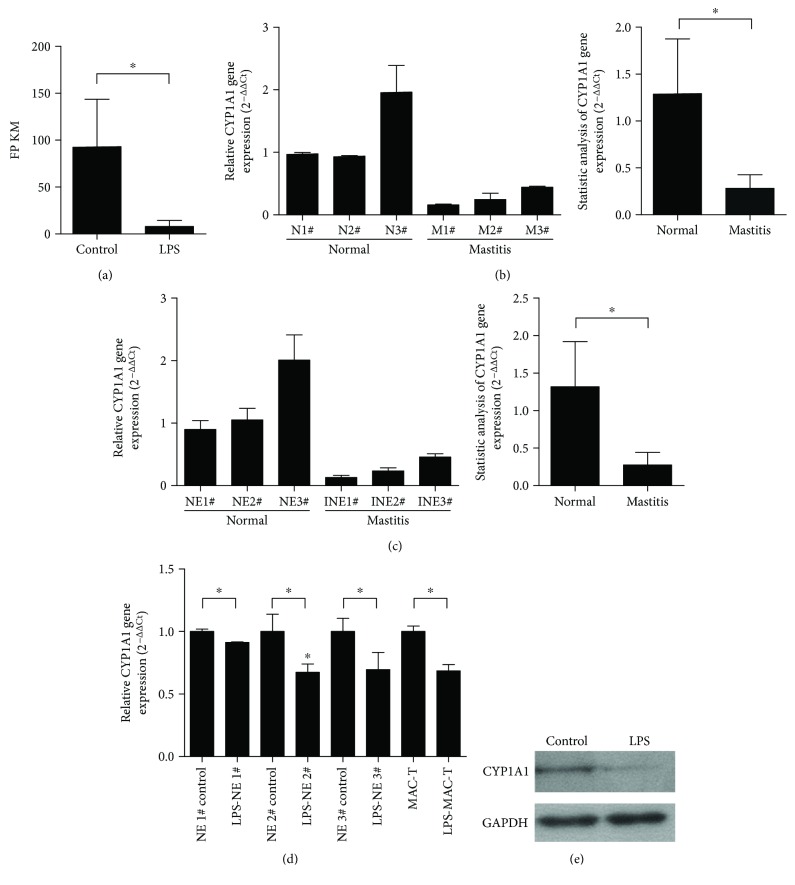
CYP1A1 mRNA and protein expression levels in bovine mammary tissue and epithelial cells. (a) CYP1A1 mRNA expression in bovine primary mammary epithelial cells treated and untreated with LPS determined using RNA-Seq (*n* = 3). Fragments per kilobase of transcript per million fragments mapped (FPKM) in RNA-Seq result were used to indicate the gene expression level. (b) CYP1A1 mRNA expression in normal and mastitic tissues analyzed using RT-qPCR. N1–3 and M1–3 indicate different individuals. (c) CYP1A1 mRNA expression in inflammatory epithelial cells (INE) extracted from mammary glands with clinical mastitis and normal epithelial cells (NE) from slaughtered dairy cows because of fractured legs during lactation was analyzed using RT-qPCR. The numbers 1, 2, and 3 indicate different individuals. (d) CYP1A1 mRNA expression in bovine primary mammary epithelial cells (NE) and MAC-T cells treated with or without LPS was analyzed using RT-qPCR. (e) CYP1A1 protein expression in MAC-T cells treated with or without LPS was analyzed by Western blot. GAPDH was used as an internal control for all experiments. Data were expressed as the mean ± SD. ^∗^ *p* < 0.05 versus control or normal.

A high-throughput sequencing analysis of the LPS-treated bovine epithelial cells was performed to screen potential genes that may mediate the infection of bovine mammary epithelial cells during mastitis. Among all the differentially expressed genes in the LPS-treated cells relative to the untreated cells, CYP1A1 has attracted our attention because of its important role in inflammation-associated diseases, such as hepatitis. High-throughput sequencing results showed that CYP1A1 expression was evidently downregulated in the LPS-induced inflammatory bovine mammary epithelial cells compared with the control (Figure 1(a)).

To further validate the CYP1A1 expression from the result of high-throughput sequencing, we extracted total mRNA from bovine normal mammary tissue and mastitic mammary tissue with clinical mastitis caused by *E. coli* and then analyzed CYP1A1 gene expression by RT-qPCR. Results showed that CYP1A1 gene expression was significantly lower in the tissue with mastitis than in the normal tissue (Figure 1(b)). Moreover, CYP1A1 gene expression was significantly downregulated in the INEs compared with the NEs (Figure 1(c)). In addition, LPS treatment downregulated the CYP1A1 expression in primary epithelial cells and immortalized MAC-T cells relative to the control (Figure 1(d)). Given that this study also investigated the functions of CYP1A1 by overexpressing it into MAC-T cells, the protein expression level of CYP1A1 in the LPS-treated MAC-T cells was also investigated using Western blot. Results showed that the protein expression level of CYP1A1 was also significantly upregulated in the LPS-induced MAC-T cells relative to the control (Figure 1(e)).

### 3.2. Construction of CYP1A1 Overexpression Plasmids and Determination of Overexpression Efficiency

**Figure 2:**
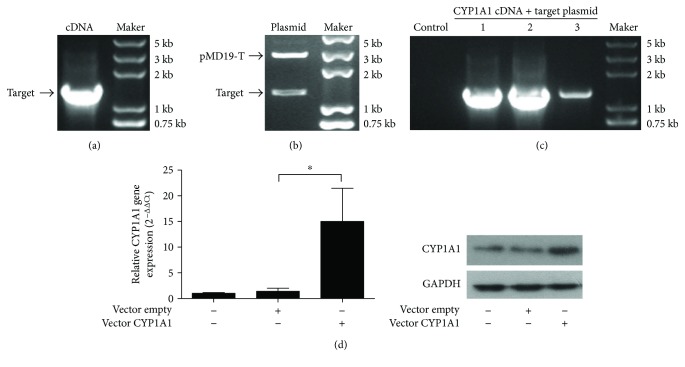
Construction of CYP1A1 overexpression plasmids and verification of overexpression efficiency. (a) Cloned full-length CYP1A1 cDNA from MAC-T cells was analyzed using PCR. (b) The dual-enzyme digestion of the plasmid of pMD19-T–CYP1A1 cDNA confirmed that CYP1A1 cDNA was accurately connected into the pMD19-T vector. (c) The efficient connection of cloned CYP1A1 cDNAs to the target plasmid was confirmed using plasmid PCR. Control: empty vector. 1, 2, and 3: target vector connected to CYP1A1 from three bacterial colonies. (d) Overexpression efficiency of CYP1A1 was verified using RT-qPCR and Western blot. GAPDH was used as an internal control. Data were expressed as the mean ± SD. ^∗^ *p* < 0.05 versus empty vector-transfected MAC-T cells.

To verify the functions of CYP1A1 in the bovine epithelial cells during bovine mastitis, we successfully cloned the full-length bovine CYP1A1 cDNAs as indicated by the PCR result (Figure 2(a)), which showed a specific band at the right base pair size of 1573 bp. The dual-enzyme digestion of the plasmid of pMD19-T–CYP1A1 cDNA confirmed that CYP1A1 cDNA was accurately connected into the pMD19-T vector (Figure 2(b)). To check the integrity and accuracy of the CYP1A1 cDNA, the cloned full-length bovine CYP1A1 cDNA was sequenced and blasted with the complete DNA sequences of encoding CYP1A1 protein in the NCBI database. Results showed that the cloned full-length bovine CYP1A1 cDNA sequences corresponded with the sequence in the NCBI database (Additional File 1: Supplemental Dataset S1). The efficient connection of the cloned CYP1A1 cDNAs with the target plasmid was confirmed by plasmid PCR (Figure 2(c)). Finally, the CYP1A1 overexpression vector was transferred into the MAC-T cell line by electroporation. After transfection for 48 h, the gene and protein expression levels of CYP1A1 were significantly upregulated in the MAC-T cell as analyzed using RT-qPCR and Western bolt (Figure 2(d)).

### 3.3. Overexpression of CYP1A1 Alleviated the Inhibitory Effects of LPS on MAC-T Cell Proliferation

**Figure 3:**
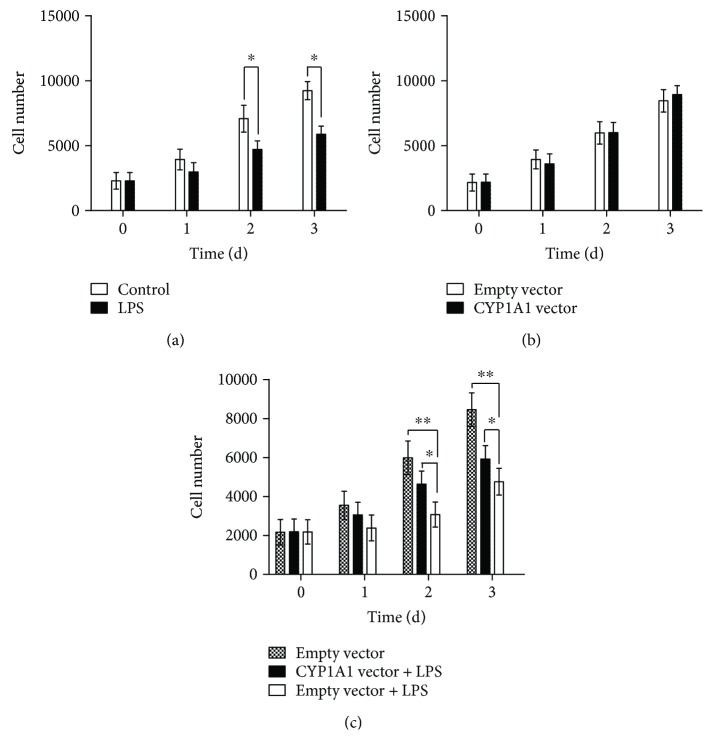
Overexpression of CYP1A1 alleviated the inhibitory effects of LPS on MAC-T cell proliferation. Cells were seeded at the same density of 2000 cells/well in 96-well plates, and the cell numbers were calculated in reference to a standard curve obtained under the same experimental conditions by using CCK-8 at indicated days. (a) Cell number of LPS-treated MAC-T cells was less than that of untreated cells at days 2 and 3, ^∗^ *p* < 0.01. (b) Cell numbers were identical between the cells with and without CYP1A1 overexpression. (c) Compared with MAC-T cells with empty vector transfection, the inhibition effects of LPS on MAC-T cell proliferation was alleviated in the cells with overexpressed CYP1A1 at the indicted day, ^∗^ *p* < 0.05, ^∗∗^ *p* < 0.01.

To investigate the effect of CYP1A1 overexpression on the proliferation of the MAC-T cells treated with LPS, we portrayed cell proliferation curves using a CCK-8 Kit. After seeding at the same density of 2000 cells/well in 96-well plates and culturing for 3 days, the number of MAC-T cells with LPS treatment was significantly less than that of the cells without LPS treatment at each indicated day (Figure 3(a)), suggesting that LPS was able to inhibit MAC-T cell proliferation. Although CYP1A1 overexpression did not significantly affect the proliferation of MAC-T cells (Figure 3(b)), its overexpression partly alleviated the inhibitory effects of LPS on MAC-T cell proliferation, as indicated in Figure 3(c). The number of MAC-T cells with CYP1A1 expression was greater than that of the empty-vector-transfected cells treated with LPS but less than that of the empty-vector-transfected cells without LPS treatment.

### 3.4. Overexpression of CYP1A1 Inhibited LPS-Induced Immune Responses in MAC-T Cells

**Figure 4:**
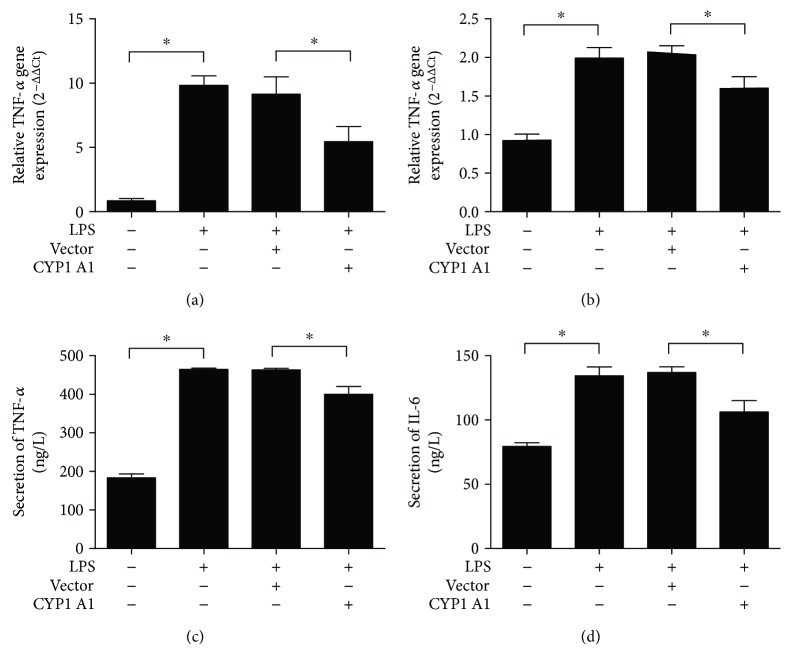
Overexpression of CYP1A1 inhibited LPS-induced immune responses in MAC-T cells. TNF-*α* (a) and IL-6 (b) gene expression levels in MAC-T cells examined using RT-qPCR. TNF-*α* (c) and IL-6 (d) protein secretion levels in MAC-T cells measured using corresponding ELISA kits. Data were expressed as the mean ± SD. ^∗^ *p* < 0.05.

Epithelia derived from bovine mammary glands with mastitis evidently express proinflammatory cytokines, such as TNF-*α* and IL-6 [19]. The present study investigated whether CYP1A1 mediates LPS-induced expression of TNF-*α* and IL-6 in MAC-T cells. Results showed that LPS indeed enhanced the mRNA expression and protein secretion levels of TNF-*α* and IL-6 in MAC-T cells and that CYP1A1 overexpression in these cells attenuated the LPS-induced increases of these two cytokines (Figure 4).

### 3.5. CYP1A1 Mediated the LPS-Induced Activation of the NF-*κ*B Signaling Pathway in MAC-T Cells

**Figure 5:**
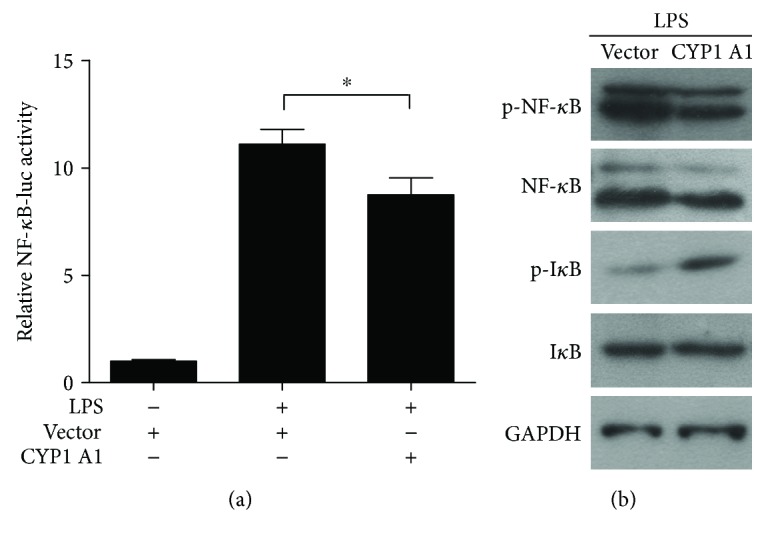
CYP1A1 overexpression mediated LPS-induced activation of the NF-*κ*B signaling pathway. (a) Activation of the NF-*κ*B signal promoter was examined using the Dual-Luciferase Reporter Assay System, ^∗^ *p* < 0.05. (b) Activation of key signal molecules of the NF-*κ*B signaling pathway was detected using Western blot.

TNF-*α* and IL-6 are key signaling molecules of the downstream NF-*κ*B signaling pathway [20]. To understand the molecular mechanism by which CYP1A1 alleviates the LPS-induced expression enhancement of TNF-*α* and IL-6 in MAC-T cells, we transfected NF-*κ*B-luc reporter DNA and control vector of pRL-TK into MAC-T cells together with a CYP1A1 plasmid to investigate the relationship among CYP1A1, NF-*κ*B, TNF-*α*, and IL-6. Results showed that LPS activated NF-*κ*B in MAC-T cells, CYP1A1 potently inhibited NF-*κ*B activation in the LPS-induced MAC-T cells, and CYP1A1 overexpression potently ameliorated the LPS-induced NF-*κ*B activation in MAC-T cells (Figure 5(a)).

In addition, Western blot analysis showed that the phosphorylation level of NF-*κ*B decreased and the phosphorylation level of I*κ*B increased in the MAC-T cells transfected with the CYP1A1 overexpression vector compared with those in the MAC-T cells transfected with the empty vector (Figure 5(b)).

## 4. Discussion

Changes in the expression levels of CYP1A1 caused by infections or inflammatory stimuli have been observed in various organs, including the liver, kidney, and brain [11]. CYP1A1 expression is upregulated in some cases but downregulated in other cases [11, 21]. In this study, CYP1A1 gene expression significantly decreased in bovine mammary tissue and extracted epithelial cells under inflammatory conditions. LPS is the major structural element of the *E. coli* cell membrane and triggers strong immune responses in various organs, including the mammary gland of bovine with coliform mastitis [22]. LPS has been widely used to establish in vitro inflammatory cell models of a series of inflammation-related diseases by treating parenchyma cells, such as human renal tubular cells [23], bovine mammary epithelial cells [19, 24], and mouse hepatocytes [25]. This study established an in vitro cell model of bovine mastitis by treating primary bovine mammary epithelial cells with LPS [19]. MAC-T cells, produced from primary bovine mammary alveolar cells by stable transfection with SV40 large T-antigen, feature optimal clonal nature, immortality, and ability to uniformly differentiate and secrete casein proteins [26]. This cell line has become the most attractive candidate to study bovine mastitis. Here, we found that LPS treatment could downregulate CYP1A1 expression in primary epithelial cells and MAC-T cells under inflammatory conditions. These findings strongly support that CYP1A1 mediates the inflammatory response of bovine mastitis.

Epithelial cells stand at the frontline resisting bacterial infections in mammary glands. When it fails, pathogens replicating in the organism release enormous toxic substances, which are always associated with bovine mastitis [27, 28]. Epithelial cells undergoing inflammation are also capable of unleashing abundant pro- and anti-inflammatory endogenous substances. Being a drug-metabolizing enzyme, CYP1A1 participates in the activation and detoxification of carcinogens and other toxicants in vertebrate animals [29, 30]. In subsequent experiments, we used LPS to treat the immortalized mammary epithelial cell line of MAC-T to investigate the functions of CYP1A1 in epithelial cells during bovine mastitis. We mainly focused on the effects of CYP1A1 on epithelial proliferation and inflammatory response indicated by the secretion of TNF-*α* and IL-6 under LPS-induced inflammatory conditions due to the following: (1) a change in the ratio of epithelial proliferation to cell death during lactation affects the persistency of lactation in dairy cattle [31]; (2) both TNF-*α* and IL-6 are important proinflammatory cytokines in inflammatory responses, regulated by NF-*κ*B signaling [32, 33], and their expressions were markedly increased in bovine primary mammary epithelial cells stimulated with LPS [24].

In this study, LPS inhibited the proliferation of MAC-T cells and induced the secretion of TNF-*α* and IL-6, whereas CYP1A1 overexpression alleviated the effects of LPS on MAC-T cells. In a previous study, LPS inhibited cell proliferation by downregulating the expression of intracellular EGFR and PDGFR via the Ras/Raf/MEK/ERK pathway [19]. Other studies indicated that CYP1A1 can promote the activation of the Ras/Raf/MEK/ERK pathway [34]. Those studies implied that CYP1A1 may promote the MAC-T cell proliferation via regulating the activation of the Ras/Raf/MEK/ERK pathway. When LPS-induced inflammatory response occurred, the expression of CYP1A1 was downregulated and thus led to the blocking of the Ras/Raf/MEK/ERK pathway in MAC-T cells; the overexpression of CYP1A1 in MAC-T cells can effectively remedy the adverse downregulation of CYP1A1 caused by LPS and promote the proliferation of MAC-T cells.

CYP1A1 gene expression is suppressed by LPS, and NF-*κ*B activation plays an important role in CYP1A1 suppression [35, 36]. The activation of NF-*κ*B classically depends on the degradation of I*κ*B. When cells are activated by a given stimulus, such as LPS, the phosphorylation of I*κ*B leads to ubiquitination by the ubiquitin ligase complex and degradation by the 26S proteasome. NF-*κ*B is separated from the complex and translocates to the nucleus, where it binds to the DNA and induces the activation of an inflammatory response [37]. Here, LPS as a proinflammatory agent activated the NF-*κ*B signaling pathway in the immortalized MAC-T cells as indicated by the result of the Dual-Luciferase Reporter Assay System of NF-*κ*B activity. This result was consistent with the previous findings that LPS can induce an inflammatory response in primary bovine mammary epithelial cells through the NF-*κ*B signaling pathway [38, 39]. Interestingly, CYP1A1 overexpression in the MAC-T cells abated the LPS-induced activation of the NF-*κ*B signaling pathway as indicated by the downregulation of pNF-*κ*B. Notably, CYP1A1 overexpression enhanced the pI*κ*B expression induced by LPS. This enhancement may be caused by the inadequate ubiquitination and degradation after the phosphorylation of I*κ*B. Considering that several types of compounds inhibited the LPS-induced inflammatory response by inhibiting the NF-*κ*B signaling pathway [39, 40], we conclude that CYP1A1 mediates the inflammatory responses of epithelial cells induced by LPS via the NF-*κ*B signaling pathway.

In summary, the results of this study suggest that CYP1A1 plays important roles in mammary epithelial cells during bovine mastitis caused by the Gram-negative bacterial cell wall component LPS. Hence, CYP1A1 may be a useful therapeutic target mitigating the injury caused by inflammatory overreaction, thus easing the worry of increasingly growing cost every year on bovine mastitis.

## Abbreviations

CYPs: Cytochrome P450 family
CYP1A1: Cytochrome P450 family 1 subfamily A member 1
IL-6: Interleukin-6
TNF-*α:*: Tumor necrosis factor-*α*
LPS: Lipopolysaccharide
PPAR-*γ:*: Peroxisome proliferator-activated receptor-*γ*.

## Supplementary Materials

Supplementary Materials Additional File 1: comparison between the cloned full-length bovine CYP1A1 cDNA sequences and the sequence in the NCBI database. Additional File 2: a list of primers for real-time PCR.
