# Supplementary material for: CYP1A1 Relieves Lipopolysaccharide-Induced Inflammatory Responses in Bovine Mammary Epithelial Cells
Source: Mediators Inflamm. 2018 Feb 28;2018:4093285. doi: 10.1155/2018/4093285 (PMC5854104; doi:10.1155/2018/4093285)
Supplement: Supplementary Materials — Additional File 1: comparison between the cloned full-length bovine CYP1A1 cDNA sequences and the sequence in the NCBI database. Additional File 2: a list of primers for real-time PCR. [file 4093285.f1.docx]

Additional File 1: Comparison between the cloned full-length bovine CYP1A1 cDNA sequences and the sequence in the NCBI database.

**
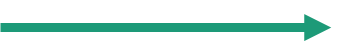
 Nhe I Forward primer**

**CYP1A1 sequencing AACTCGGTACGCGCGGATCTTCCAGAGATTCTAGCTAGCGGATCATGTTTCCTGTGTTTGGACTCCCC**

**CYP1A1 NCBI database ATGTTTCCTGTGTTTGGACTCCCC**

**--------------------------------------------**************************

**CYP1A1 sequencing ATCCCCATCTCGGCCACAGAACTTCTCCTGGCCTCTGCCGTCTTCTGCCTGGTATTCTGGGTGGTCAG**

**CYP1A1 NCBI database ATCCCCATCTCGGCCACAGAACTTCTCCTGGCCTCTGCCGTCTTCTGCCTGGTATTCTGGGTGGTCAG**

************************************************************************

**CYP1A1 sequencing GACCTGGCGGCCTCGGGTCCCTCAAGGCCTGAAGAGTCCCCCGGAGCCCTGGGGCTGGCCCCTGCTCG**

**CYP1A1 NCBI database GACCTGGCGGCCTCGGGTCCCTCAAGGCCTGAAGAGTCCCCCGGAGCCCTGGGGCTGGCCCCTGCTCG**

************************************************************************

**CYP1A1 sequencing GGCACATGCTGATGTTGGGGAAGAACCCACACGTGGTCCTGTCGCAGCTGAGCCAGCGCTATGGGGAC**

**CYP1A1 NCBI database GGCACATGCTGATGTTGGGGAAGAACCCACACGTGGTCCTGTCGCAGCTGAGCCAGCGCTATGGGGAC**

************************************************************************

**CYP1A1 sequencing GTGCTGCAGATCCGCATTGGCTGCACACCCGTGCTGGTGCTCAGCGGCCTGGACACCGTCCGGCAGGC CYP1A1 NCBI database GTGCTGCAGATCCGCATTGGCTGCACACCCGTGCTGGTGCTCAGCGGCCTGGACACCGTCCGGCAGGC**

************************************************************************

**CYP1A1 sequencing CCTCGTGCGGCAGGGCGATGATTTCAAGGGCCGGCCCGACCTCTACAGCTTCACCTTGATCACTAACG CYP1A1 NCBI database CCTCGTGCGGCAGGGCGATGATTTCAAGGGCCGGCCCGACCTCTACAGCTTCACCTTGATCACTAACG**

************************************************************************

**CYP1A1 sequencing GCCAGAGCATGACCTTCAACCCAGACTCTGGACCGGTGTGGGCTGCCCGACGACGCCTGGCCCAGAAT CYP1A1 NCBI database GCCAGAGCATGACCTTCAACCCAGACTCTGGACCGGTGTGGGCTGCCCGACGACGCCTGGCCCAGAAT**

************************************************************************

**CYP1A1 sequencing GCTCTGAAGAGTTTCTCCACTGCCTCAGACCCGGCATCCTCATCCTCTTGCTATCTGGAAGAGCATGT CYP1A1 NCBI database GCTCTGAAGAGTTTCTCCACTGCCTCAGACCCGGCATCCTCATCCTCTTGCTATCTGGAAGAGCATGT**

************************************************************************

**CYP1A1 sequencing GAACAAGGAGGCCAAGTACCTCCTGGGCAAGTTCCAAGAGCTGATGTCAGGGCCTGGGCGCTTTGACC CYP1A1 NCBI database GAACAAGGAGGCCAAGTACCTCCTGGGCAAGTTCCAAGAGCTGATGTCAGGGCCTGGGCGCTTTGACC**

************************************************************************

**CYP1A1 sequencing CCTACAGGTATATAGTGGTGTCAGTGGCCAATGTCATCTGTGCCATATGCTTTGGCCGGCGCTATGAC CYP1A1 NCBI database CCTACAGGTATATAGTGGTGTCAGTGGCCAATGTCATCTGTGCCATATGCTTTGGCCGGCGCTATGAC**

************************************************************************

**CYP1A1 sequencing CACAATGACCAAGAGTTTCTTAGCCTCGTCAACCTGAGTAATGAGTTTGGGGAGATAACTGCCTCCGG CYP1A1 NCBI database CACAATGACCAAGAGTTTCTTAGCCTCGTCAACCTGAGTAATGAGTTTGGGGAGATAACTGCCTCCGG**

************************************************************************

**CYP1A1 sequencing GAACCCATCTGACTTCATCCCTGTCCTCCGTTACCTGCCCAACACTGCCCTGGACCTCTTCAAGGACC CYP1A1 NCBI database GAACCCATCTGACTTCATCCCTGTCCTCCGTTACCTGCCCAACACTGCCCTGGACCTCTTCAAGGACC**

************************************************************************

**CYP1A1 sequencing TGAATCAGAGGTTCTACGTCTTTGTACAGAAGATAGTCAAGGAACACTATAAAACGTTTGAGAAGGGT CYP1A1 NCBI database TGAATCAGAGGTTCTACGTCTTTGTACAGAAGATAGTCAAGGAACACTATAAAACGTTTGAGAAGGGT**

************************************************************************

**CYP1A1 sequencing CACATCCGGGACATCACAGACAGCCTGATTGAGCACTGTCAGGACAAGAGGCTGGACGAGAATGCCAA CYP1A1 NCBI database CACATCCGGGACATCACAGACAGCCTGATTGAGCACTGTCAGGACAAGAGGCTGGACGAGAATGCCAA**

************************************************************************

**CYP1A1 sequencing TATCCAGCTGTCGGATGAGAAGATCATTAATGTTGTCATAGACCTCTTTGGAGCCGGGTTTGACACAG CYP1A1 NCBI database TATCCAGCTGTCGGATGAGAAGATCATTAATGTTGTCATAGACCTCTTTGGAGCCGGGTTTGACACAG**

************************************************************************

**CYP1A1 sequencing TCACAACTGCCCTTTCCTGGAGCCTCCTGTACCTGGTGACAAGCCCCAGGGTGCAAAAAAAGATTCAG CYP1A1 NCBI database TCACAACTGCCCTTTCCTGGAGCCTCCTGTACCTGGTGACAAGCCCCAGGGTGCAAAAAAAGATTCAG**

************************************************************************

**CYP1A1 sequencing GAGGAGCTGGACACAGTGATTGGCAGGGCGCGGCGGCCCCGGCTCTCTGACAGACCCCAGCTGCCCTA CYP1A1 NCBI database GAGGAGCTGGACACAGTGATTGGCAGGGCGCGGCGGCCCCGGCTCTCTGACAGACCCCAGCTGCCCTA**

************************************************************************

**CYP1A1 sequencing TTTGGAGGCCTTTATCCTGGAGACCTTCCGACACTCCTCCTTTGTCCCCTTCACCATCCCACACAGTA CYP1A1 NCBI database TTTGGAGGCCTTTATCCTGGAGACCTTCCGACACTCCTCCTTTGTCCCCTTCACCATCCCACACAGTA**

************************************************************************

**CYP1A1 sequencing CCACAAGAGACAGCAATCTGAACGGCTTTTACATCCCCAAGGGGCGCTGTGTCTTTGTGAACCAGTGG CYP1A1 NCBI database CCACAAGAGACAGCAATCTGAACGGCTTTTACATCCCCAAGGGGCGCTGTGTCTTTGTGAACCAGTGG**

************************************************************************

**CYP1A1 sequencing CAGATCAACCATGACCAGAAGCTCTGGGAGGATCCATCTGAGTTCCGGCCAGAACGGTTTCTCACTGC CYP1A1 NCBI database CAGATCAACCATGACCAGAAGCTCTGGGAGGATCCATCTGAGTTCCGGCCAGAACGGTTTCTCACTGC**

************************************************************************

**CYP1A1 sequencing TGATGGCACCATCAACAAAGTACTGAGTGAGAAGGTGATTATTTTCGGCTTGGGCAAGCGGAAGTGCA CYP1A1 NCBI database TGATGGCACCATCAACAAAGTACTGAGTGAGAAGGTGATTATTTTCGGCTTGGGCAAGCGGAAGTGCA**

************************************************************************

**CYP1A1 sequencing TCGGTGAGACCATTGCCCGCTTGGAGGTCTTTCTCTTCTTGGCCATCCTGCTGCATCAGGTGGAATTC CYP1A1 NCBI database TCGGTGAGACCATTGCCCGCTTGGAGGTCTTTCTCTTCTTGGCCATCCTGCTGCATCAGGTGGAATTC**

************************************************************************

**CYP1A1 sequencing TGTGTGACCCCGGGTGTGAAGGTGGACATGACCCCCGTGTACGGGCTGACCATGAAGTACGCCCGCTG CYP1A1 NCBI database TGTGTGACCCCGGGTGTGAAGGTGGACATGACCCCCGTGTACGGGCTGACCATGAAGTACGCCCGCTG**

************************************************************************


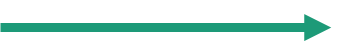
 **Reverse primer Not I**

**CYP1A1 sequencing TGAGCACTTTCAGGCGCACATGCGCTCTTAGGCGGCCGCAATCTCTAGAGGATCCCCGGGTACCGAGC**

**CYP1A1 NCBI database TGAGCACTTTCAGGCGCACATGCGCTCTTAG**

*********************************-------------------------------------**

**CYP1A1 sequencing TCGAATCGTAATCAGTCATTT**

**CYP1A1 NCBI database**

**-------------------**

Additional File 2. A List of Primers for real time PCR.

| Gene | Accession number | Forward primer | Reverse primer |
| --- | --- | --- | --- |
| CYP1A1 | XM_005192890.3 | GGTTTTTCTCTTCTTGGCCATCC | TTTTAGGCTCCCCTGGGCTA |
| TNF-α | GU129693.1 | GTTCTCCCCATGACACCACCTG | GGGAGAAGAGAGTCAGACAGGC |
| IL-6 | NM_173923.2 | ACAGCTATGAACTCCCGCTT | TCGACCATGCGCTTAATGAGA |
| GAPDH | NM_001034034.2 | GGTCACCAGGGCTGCTTTTA | CCAGCATCACCCCACTTGAT |
